# Burnout syndrome among medical residents: A systematic review and meta-analysis

**DOI:** 10.1371/journal.pone.0206840

**Published:** 2018-11-12

**Authors:** Hugo Rodrigues, Ricardo Cobucci, Antônio Oliveira, João Victor Cabral, Leany Medeiros, Karen Gurgel, Tházio Souza, Ana Katherine Gonçalves

**Affiliations:** 1 Health Science Postgraduate Program, Universidade Federal do Rio Grande do Norte, Natal, Brazil; 2 Department of Obstetrics and Gynecology, Potiguar University, Natal, Brazil; 3 Department of Pharmacy, Universidade Federal do Rio Grande do Norte, Natal, Brazil; 4 Medical student, Universidade Federal do Rio Grande do Norte, Natal, Brazil; 5 Medical resident at Family Medicine Program, Hospital Santa Marcelina, São Paulo, Brazil; 6 Medical resident at Obstetrics and Gynecology Program, Universidade Federal do Rio Grande do Norte, Natal, Brazil; 7 Department of Obstetrics and Gynecology, Universidade Federal do Rio Grande do Norte, Natal, Brazil; Medical University Hospital Tuebingen, GERMANY

## Abstract

**Background:**

Burnout is a psychological syndrome that is very common among medical residents. It consists of emotional exhaustion (EE), depersonalization (DP) and reduced personal accomplishment (PA).

**Objective:**

To estimate burnout among different medical residency specialties.

**Methods:**

A systematic review with meta-analysis was performed following the Preferred Reporting Items for Systematic Reviews and Meta-Analyses (PRISMA) guidelines. A search of bibliographic databases and grey literature was conducted, from inception to March 2018. The following databases were accessed: Embase, PubMed, Web of Science, Google Scholar and Scopus, and 3,575 studies were found. Methodological quality was evaluated by *Agency for Healthcare Research and Quality Methodology Checklist for Cross-Sectional/Prevalence Study*. In the final analysis, 26 papers were included. Their references were checked for additional studies, but none were included.

**Results:**

4,664 medical residents were included. High DP, EE and low PA proportions were compared. Specialties were distributed into three groups of different levels of burnout prevalence: general surgery, anesthesiology, obstetrics/gynecology and orthopedics (40.8%); internal medicine, plastic surgery and pediatrics (30.0%); and otolaryngology and neurology (15.4%). Overall burnout prevalence found for all specialties was 35.7%.

**Conclusion:**

The prevalence of burnout syndrome was significantly higher among surgical/urgency residencies than in clinical specialties.

**PROSPERO registration:**

CRD42018090270.

## Introduction

First described in 1974,[1] burnout syndrome is a psychological syndrome arising from a continued response to chronic interpersonal stressors while at work.[2] A generic description of burnout defines it as a state of physical and mental exhaustion related to caregiving activities or work.[1,3]

Work-related stress among healthcare professionals has become a serious health problem for workers and the world economy.[4] The syndrome among both doctors in practice and in training, has reached epidemic levels, with a prevalence near to or exceeding 50%.[5–8] Additionally, it has a notable economic impact, since the cost of replacing a physician in the workplace is up to 2–3 times his/her annual salary.[4]

Situations of emotional exhaustion and irritability in the work environment could lead to the development of psychiatric problems, with an emphasis on burnout, which is characterized by its subdimensions, being emotional exhaustion, depersonalization and diminished personal accomplishment.[9,10]

The consequences of burnout are potentially severe for caregivers, patients and health institutions, and include the risk of medical errors, depression, and adverse effects on patient safety.[3] A recent systematic review, including 20 articles, suggests that burnout affects primary healthcare providers (mainly nurses, but also physicians and pharmacists) and leads to high job stress, intense time pressure and workload as well as lack of organizational support.[11] The syndrome also affects patient satisfaction,[12] and along with personal distress, it has been related to self-reported suboptimal patient care practices among residents across numerous medical specialties.[8,13,14]

Focusing on its subdimensions, emotional exhaustion (EE), refers to feelings of overload and depletion of emotional resources; depersonalization (DP), is the negative response to other people, such as colleagues and patients, in a cynical and isolated way; and reduced personal accomplishment (PA), occurs when the subject feels less competent in his/her role.[9,10]

In this context, the Maslach Burnout Inventory (MBI)[15] is the most commonly used self-completion questionnaire for assessing burnout, as it presents greater validity and increased reliability concerning the multiple dimensions of the syndrome than other less common instruments.[9] It was designed to evaluate the three subdimensions, and consists of 22 items divided into three subscales. The EE subscale evaluates the complaints about feeling on edge and exhausted by work. The DP subscale measures impersonal responses and lack of empathy during professional activity, while the PA subscale evaluates the feelings of competence and achievement of success at work.

Resident physicians must develop specific skills in their chosen area during their medical residency in order to maintain quality of patient care.[16] During this period they are subjected to sleep deprivation, high workload and unsatisfactory salaries,[17] as well as taking on many responsibilities in their workplaces.[18,19] This combination of factors makes them vulnerable to the development of burnout,[18] leading to interference with the individual's ability to sort through diagnostic dilemmas, establish rapport, as well as work through complex treatment decision-making.[3] Studies have suggested that residents may experience adverse mental health and work performance, with a high prevalence of the syndrome.[20]

Published data in the scientific literature on burnout syndrome in residents are limited to one-specialty evaluation. Information that analyzes the prevalence of the syndrome in multiple specialties simultaneously would help indicate which residents are more susceptible to this syndrome. A comprehensive search was conducted in 2005, which included 19 studies, but it did not follow strict criteria and, what is more, it is more than ten years old.[21]

The goal of this systematic review with meta-analysis is to summarize the published studies and to estimate burnout syndrome prevalence among different medical resident specialties, as well as to point out the medical specialties most affected by the syndrome.

## Methods

This systematic review followed the Preferred Reporting Items for Systematic Reviews and Meta-Analyses (PRISMA)[22] and was registered with the International Prospective Register of Systematic Reviews (PROSPERO reference CRD42018090270).

### Data sources and search strategy

A search was conducted using a combination of free-text and medical subject heading (MeSH) search terms, text words and keywords based on each database characteristic focusing on synonyms of burnout syndrome and medical residents.

Accessing the MeSH Database, the following heading related to the burnout syndrome was found: *Burnout*, *Professional–an excessive stress reaction to one’s occupational or professional environment*. *It is manifested by feelings of emotional and physical exhaustion coupled with a sense of frustration and failure*. In regard to medical residents, the MeSH heading is *Internship and Residency–Programs of training in MEDICINE and medical specialties offered by hospitals for graduates of MEDICINE to meet the requirements established by accrediting authorities*.

Thus, the search strategy utilized was *((((((medical resident) OR resident) OR residency training) OR residency) OR (internship and residency))) AND (((burnout) OR burnout syndrome) OR professional burnout*. The following databases were accessed: Embase, PubMed, Web of Science, Google Scholar and Scopus, which yielded 3,575 citations. The above search strategy was used in PubMed, but equivalent synonyms were utilized in each particular database. Electronic searches were made for articles published from January, 1974, when burnout was first described,[1] to March 2018. No language restriction was imposed. No medical librarian was recruited for the search.

### Evaluation of the methodological quality of the primary studies

An evaluation of the methodological quality/risk of bias of the primary cross-sectional studies was performed with the instrument Agency for Research and Health Quality (AHRQ) Methodology Checklist for Cross-sectional Study/Prevalence.[23] The AHRQ checklist consists of 11 items, with classifications of ‘yes’, ‘no’, or “unclear’. In this study, the articles are classified as excellent (ten or more items with a ‘yes’ response); ‘good’ (seven to nine ‘yes’ answers); ‘weak’ (from four to six ‘yes’ responses) and ‘poor’ methodological quality (from one to three ‘yes’ answers).

### Inclusion and exclusion criteria

The following inclusion criteria were defined: study design—cross-sectional/survey studies; population—medical residents during their specialization training programs; intervention—22 item-MBI version[15] was used as measurement instrument; controls—medical specialties were compared among themselves.; outcome—whether criteria for burnout syndrome, or any of its subdimensions, were present (positivity) according to the MBI; the subdimensions cutoff points adopted—low EE ≤ 18, high EE ≥ 27; low DP ≤ 5, high DP ≥ 10; and high PA ≥ 40, low PA ≤ 33.[24–27]; burnout risk defined as high DP and/or EE cutoffs (low PA was not an obligatory criterion); publication time after 1974, and; ‘excellent’ and ‘good’ quality studies according to Agency for Healthcare Research and Quality (AHRQ) Methodology Checklist for Cross-Sectional/Prevalence Study.[23]

There have been a significant number of studies that have applied shortened versions of the MBI, however, these have included confusing variables and thus have not allowed proper analysis of the subdimensions. Since the full version of the MBI is the most robust and complete burnout measurement tool, others versions were not accepted in this research.

Exclusion criteria were: studies not submitted to and approved by Ethical Committees (or similar); longitudinal studies; results not specified by each individual specialty, and; study population as subgroups among medical residents themselves.

Intervention studies were also excluded, even though there was the possibility that they would provide additional data; it was considered that they would represent a source of heterogenous data and thus would be a conceptual error.

### Selection of articles for review

**Fig 1:**
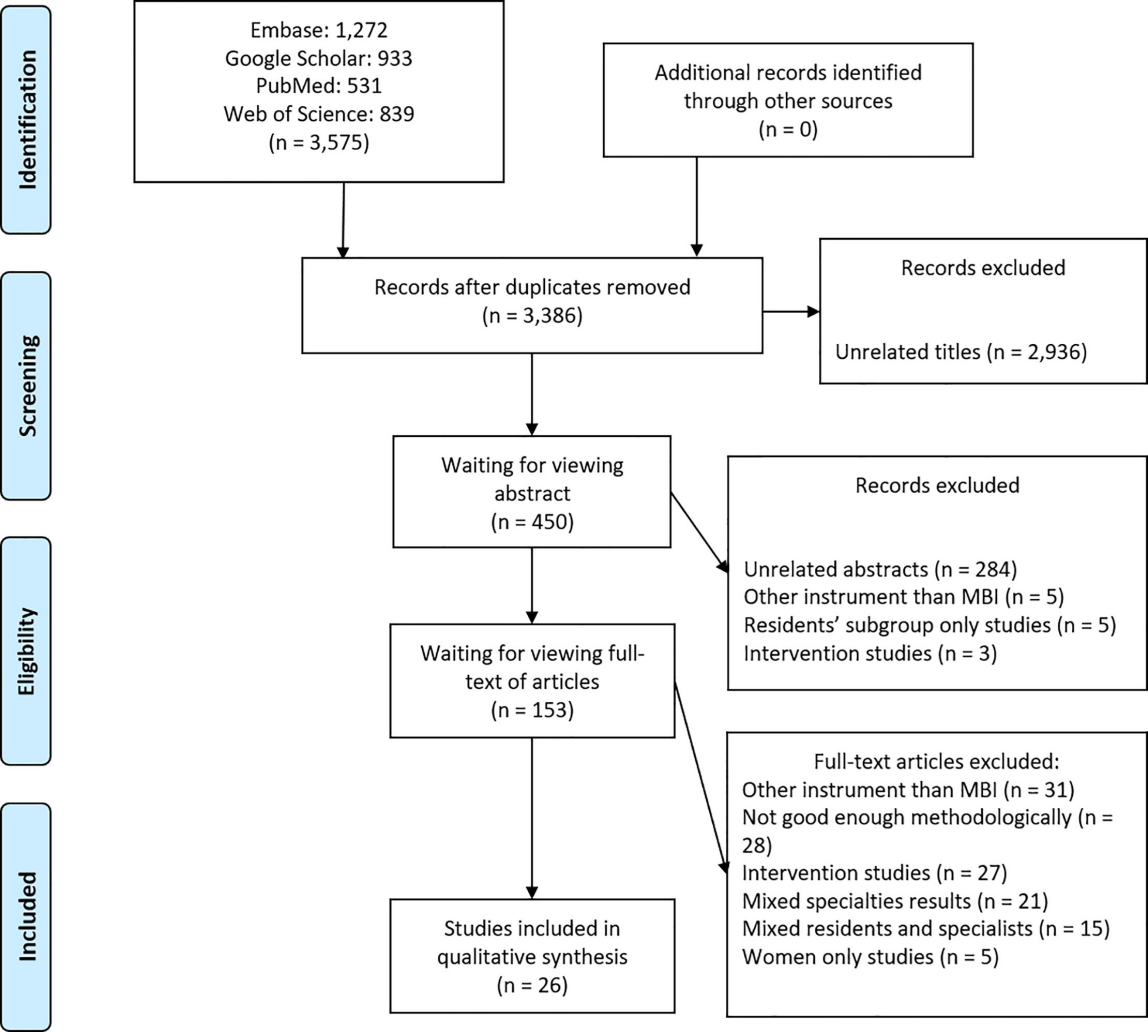
Article selection. Selection process in accordance with the PRISMA (Preferred Reporting Items for Systematic Reviews and Meta-analyses).[22].

The initial review of the articles was based on analysis of the titles. From the total number of retrieved articles (n = 3,575), 189 were excluded because they were found in more than one database, leaving 3,386 studies. At the screening stage, 2,936 papers were excluded because their titles were too distant from the research theme. The total of approved titles was 450. A selection of these articles from the contents of their abstracts excluded 297 for one or more of the following reasons: (1) unrelated to the research theme; (2) a questionnaire other than the MBI was used; (3) not cross-sectional/survey studies or (4) the study population was restricted to specific subgroups among medical residents (for instance, only male, or only female). The remaining 153 articles were read entirely and 26 were selected to be included in the systematic review, after being considered ‘excellent’ and/or ‘good’ methodological studies. The other 127 papers were also excluded because they were intervention studies; did not present enough research data; had mixed specialty results or had mixed resident and specialist groups. (Fig 1)

The included papers also had their references researched for additional studies that could be included on the systematic review, but no others were found. Four researchers (LFM, HMR, JVSC, and AKG) conducted the process of selecting articles, independently. Disagreements were solved by consensus.

### Data extraction

The data were carefully evaluated and extracted independently from all the eligible publications. Data retrieved from the studies included author, publication year, country, specialties evaluated, population size, scores on each MBI dimension and the overall burnout value as shown in Table1.

**Table 1:** Characterization of systematic review included studies.

Author	Publication year	Country	Specialties	Sample (n)	Depersonalization % (n)			Emotional Exhaustion % (n)			Personal Accomplishment % (n)			Burnout % (n)
					low	medium	high	Low	medium	High	low	medium	high	
Akturk[29]	2015	Turkey	Radiation Oncology	**45**	37.8 (17)	35.6 (16)	26.7 (12)	15.6 (7)	35.6 (16)	48.9 (22)	13.3 (6)	17.8 (8)	68.9 (31)	^a^
														
Aldrees[30]	2015	Saudi Arabia	Otolaryngology	85	21.2 (18)	23.6 (20)	55.3 (47)	14.1 (12)	23.5 (20)	62.3 (53)	56.5 (48)	27.1 (23)	16.5 (14)	44.7 (38)
Golub[25]	2007	U.S.A.	Otolaryngology	514	25.1 (129)	22.0 (113)	52.9 (272)	37.9 (195)	29.0 (149)	33.1 (170)	21.6 (111)	30.3 (156)	48.0 (247)	9.92 (51)
			Subtotal	**599**	25.6 (147)	22.2 (133)	53.3 (319)	34.6 (207)	28.2 (169)	37.2 (223)	26.5 (159)	29.9 (179)	43.6 (261)	14.9 (89)
														
Al-Ma’mari[31]	2016	Canada	Obstetrics and Gynecology	143	^a^	^a^	64.3 (92)	^a^	^a^	50.3 (72)	24.5 (35)	^a^	^a^	73.4 (105)
Castelo-Branco[32]	2006	Spain	Obstetrics and Gynecology	109	^a^	^a^	53.2 (58)	^a^	^a^	21.1 (23)	^a^	^a^	^a^	57.8 (63)
Garza[33]	2004	U.S.A.	Obstetrics and Gynecology	136	^a^	^a^	47.1 (64)	^a^	^a^	38.2 (52)	19.1 (26)	^a^	^a^	17.6 (24)
Govardhan[34]	2012	U.S.A.	Obstetrics and Gynecology	49	18.4 (9)	28.6 (14)	53.1 (26)	20.4 (10)	22.4 (11)	57.1 (28)	38.8 (19)	32.6 (16)	28.6 (14)	14.3 (7)
Rua[35]	2013	France	Obstetrics and Gynecology	36	36.1 (13)	33.3 (12)	30.6 (11)	47.2 (17)	33.3 (12)	19.4 (7)	33.3 (12)	55.6 (20)	11.1 (4)	36.1 (13)
			Subtotal	**473**	25.9 (22)	30.6 (26)	53.1 (251)	31.8 (27)	27.1 (23)	38.5 (182)	25.3 (92)	42.3 (36)	21.2 (18)	44.9 (212)
														
de Andrade[36]	2016	Brazil	Pediatrics	32	40.6 (13)	34.4 (11)	25.0 (8)	46.9 (15)	34.3 (11)	18.7 (6)	3.13 (1)	59.4 (19)	37.5 (12)	18.7 (6)
Olson[37]	2015	U.S.A.	Pediatrics	45	^a^	^a^	24.4 (11)	^a^	^a^	26.7 (12)	8.89 (4)	^a^	^a^	40.0 (18)
			Subtotal	**77**			24.7 (19)			18.0 (23.38)	6.49 (5)			31.2 (24)
														
Arora[38]	2014	Australia	Orthopedics	51	19.6 (10)	45.1 (23)	35.3 (18)	19.6 (10)	35.3 (18)	45.1 (23)	33.3 (17)	33.3 (17)	33.3 (17)	52.9 (27)
Simons[39]	2016	U.S.A.	Orthopedics	27	40.7 (11)	22.2 (6)	37.0 (10)	26.0 (7)	44.4 (12)	29.6 (8)	26.0 (7)	33.3 (9)	40.7 (11)	40.7 (11)
			Subtotal	**78**	26.9 (21)	37.2 (29)	35.1 (28)	21.8 (17)	38.5 (30)	39.7 (31)	30.8 (24)	33.3 (26)	35.9 (28)	48.7 (38)
Chaput[40]	2015	France	Plastic Surgery	**52**	59.6 (31)	15.4 (8)	25.0 (13)	63.5 (33)	23.1 (12)	13.5 (7)	46.1 (24)	21.1 (11)	30.8 (16)	28.8 (15)
Elmore[41]	2016	U.S.A.	General Surgery	664	20.9 (139)	29.2 (194)	49.8 (331)	19.7 (131)	23.5 (156)	56.8 (377)	16.1 (107)	37.5 (249)	46.4 (308)	34.0 (226)
Malik[42]	2016	Pakistan	General Surgery	133	24.8 (33)	25.6 (34)	49.6 (66)	16.5 (22)	33.1 (44)	50.4 (67)	53.4 (71)	26.3 (35)	20.3 (27)	57.9 (77)
			Subtotal	**797**	21.6 (172)	28.6 (228)	49.1 (397)	19.2 (153)	25.1 (200)	55.7 (444)	22.3 (178)	35.6 (284)	42.0 (335)	38.0 (303)
Jalili[43]	2013	Iran	Urgency and Emergency	**165**	^a^	^a^	39.4 (65)	^a^	^a^	37.0 (61)	46.1 (76)	^a^	^a^	^a^
Lebensohn[44]	2013	U.S.A.	Family Medicine	**168**	50.6 (85)	25.6 (43)	23.8 (40)	58.3 (98)	28.0 (47)	13.7 (23)	^a^	^a^	^a^	^a^
Legassie[45]	2008	Canada	Internal Medicine	48	29.2 (14)	35.4 (17)	35.4 (17)	39.6 (19)	31.2 (15)	29.1 (14)	27.1 (13)	41.7 (20)	31.2 (15)	12.5 (6)
Olson[46]	2014	U.S.A.	Internal Medicine	78	^a^	^a^	^a^	^a^	^a^	^a^	^a^	^a^	^a^	52.6 (41)
Sajjadi[47]	2017	Canada	Internal Medicine	43	21.0 (9)	32.6 (14)	46.5 (21)	11.6 (5)	39.5 (17)	48.8 (21)	32.6 (14)	34.9 (15)	32.6 (14)	20.9 (9)
Shapiro[48]	2015	U.S.A.	Internal Medicine	94	^a^	^a^	50.0 (47)	^a^	^a^	45.7 (43)	77.7 (73)	^a^	^a^	33.0 (31)
Adám[49]	2009	Hungary	Internal Medicine	43	^a^	^a^	41.9 (18)	^a^	^a^	25.6 (11)	97.7 (42)	^a^	^a^	^a^
			Subtotal	**306**	25.3 (23)	34.1 (31)	47.7 (102)	26.4 (24)	35.2 (32)	39.0 (89)	62.3 (142)	38.5 (35)	31.9 (29)	33.1 (87)
McNeeley[50]	2013	U.S.A.	Radiology	**266**	50.7 (135)	^a^	49.2 (131)	47.0 (124)	^a^	53.4 (142)	^a^	^a^	^a^	^a^
De Oliveira[51]	2013	U.S.A.	Anesthesiology	**1,417**	^a^	^a^	^a^	^a^	^a^	^a^	^a^	^a^	^a^	40.6 (575)
Zis[52]	2015	Greece	Neurology	**116**	^a^	^a^	^a^	^a^	^a^	^a^	^a^	^a^	^a^	18.1 (21)
Waldman[53]	2009	Argentina	Cardiology	**105**	^a^	^a^	72.4 (76)	^a^	^a^	67.6 (71)	10.5 (11)	^a^	51.4 (54)	^a^
** **	** **	** **	**Total**	**4,664**	**34.6 (1051)**	**27.0 (525)**	**47.6 (1453)**	**31.9 (706)**	**27.7 (540)**	**43.0 (1313)**	**28.05 (704)**	**35.6 (598)**	**41.6 (784)**	**35.7 (1364)**

Extracted data from approved studies.

^a^Data not informed.

### Statistical analysis

The data were analyzed with STATA 12.0 [28]. Prevalence standard errors were calculated using the standard formula for proportions: sqrt[p*(1 –p)/n]; Heterogeneity across studies in the proportion of medical residents presenting with burnout syndrome, high DP, high EE and low PA was tested with the chi-square and the proportion of total variation across studies attributable to heterogeneity was estimated by the *I*^*2*^ statistic. As there was evidence of significant heterogeneity across studies, the point estimates from each study were combined using a random effects meta-analysis model with the overall estimate obtained with the DerSimonian-Laird method. Sources of heterogeneity across studies were examined with meta-regression. Publication bias and small study effects were assessed with the Egger test.

## Results

**Fig 2:**
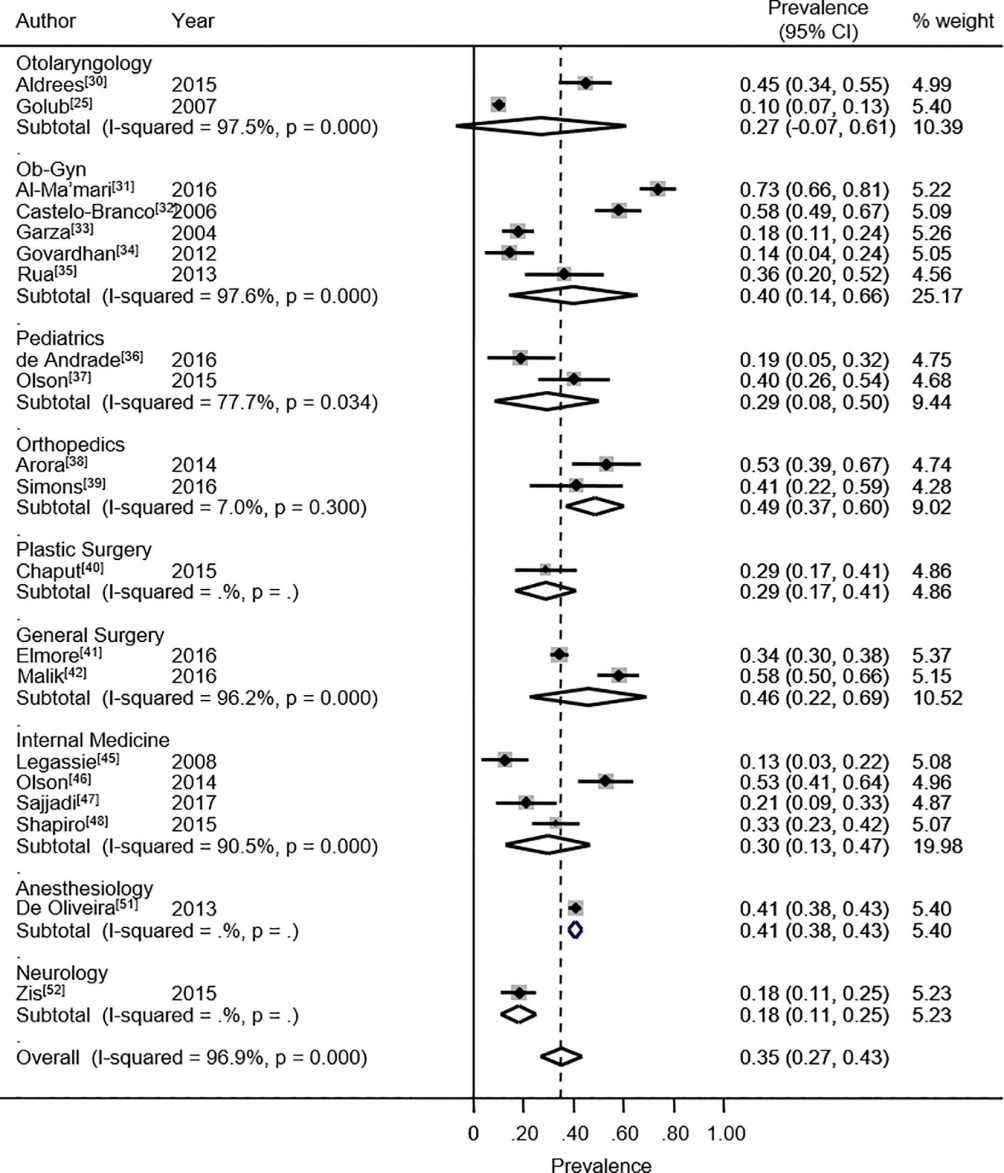
Burnout prevalence. Forest plot of prevalence of burnout syndrome among medical and surgical residents.

The 26 approved studies involved 4,664 medical residents. The design features of the selected studies are indicated in Table 1. The overall burnout prevalence found for all specialties was 35.1% (95% CI: 26.8% - 43.5%). This estimate was based on 20 studies. The heterogeneity chi-squared was 609.75 (p<0.001) with an *I*^2^ statistic of 96.9%. This comparison of the proportion of residents presenting burnout between all types of specialties suggests that the specialties are distributed into three groups with different levels of the syndrome: a group composed of general surgery,[41,42] anesthesiology,[51] obstetrics and gynecology,[31–35] and orthopedics[38,39] with a high prevalence of 42.5%; a group formed by internal medicine,[45–49] plastic surgery[40] and pediatrics,[36,37] with a moderate prevalence of 29.4%; and finally a group including otolaryngology[25,30] and neurology,[16] with a low burnout syndrome prevalence of 23.5% (Fig 2). However, no statistically significant difference was found by meta-regression (p = 0.17).

**Fig 3:**
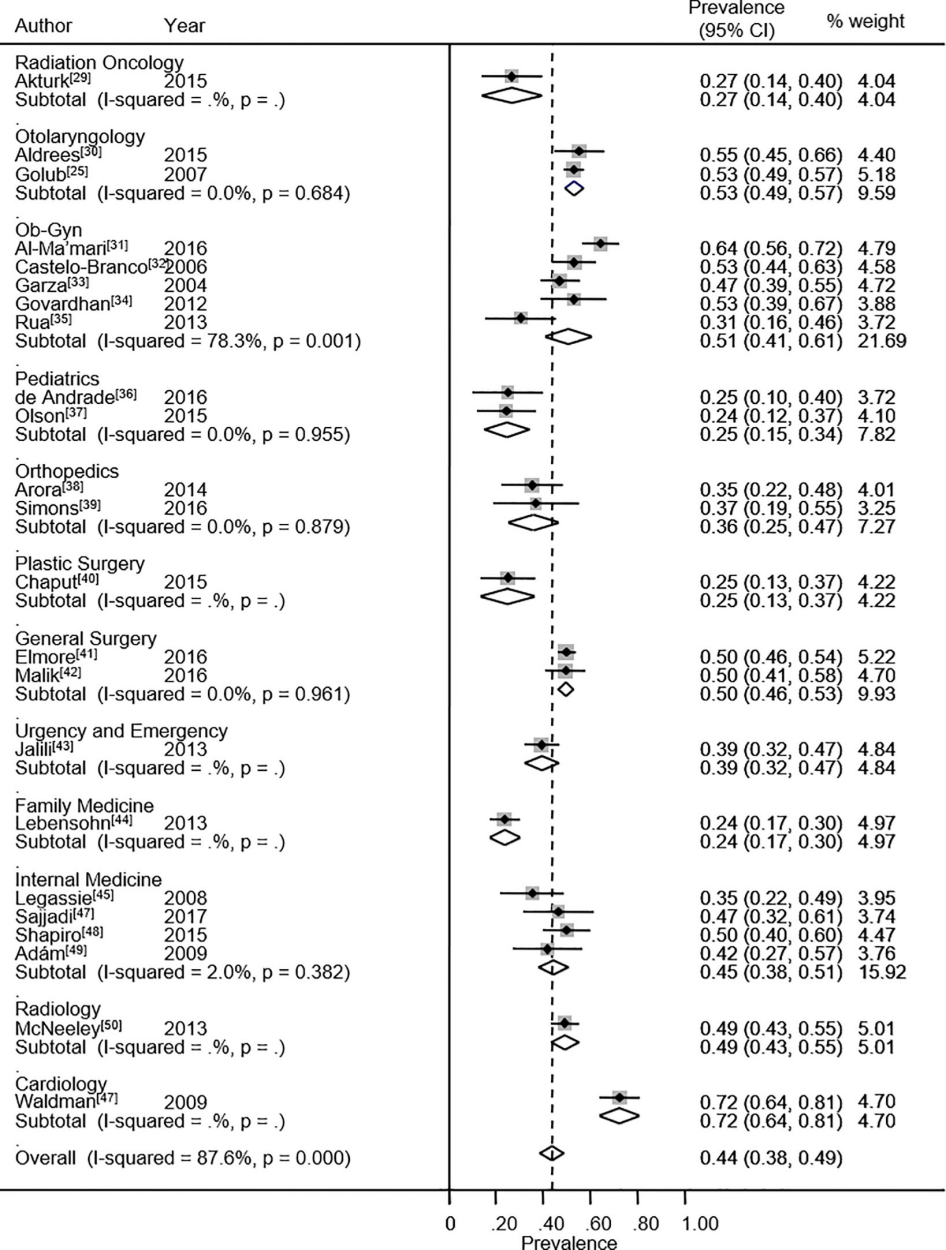
Depersonalization. Forest plot of prevalence of high depersonalization among medical and surgical residents.

Concerning subdimensions, prevalence rates of high DP were reported in 23 studies. The heterogeneity chi-squared was 177.4 (p<0.001) with an *I*^2^ statistic of 87.6%. The meta-analytic prevalence estimate of high DP for all specialties was 43.6% (95% CI: 38.4% - 48.9%). The highest DP values were found in cardiology (defined as a medical residency for the purposes of the systematic review, although it is defined as a fellowship in the U.S.A),[53] otolaryngology with 53.3%[25,30] and obstetrics and gynecology with 50.6%.[31–35] (Fig 3). The specialties showing the highest percentages of low DP were plastic surgery (59.6%),[40] radiology (50.8%)[50] and family medicine (50.6%).[44]

**Fig 4:**
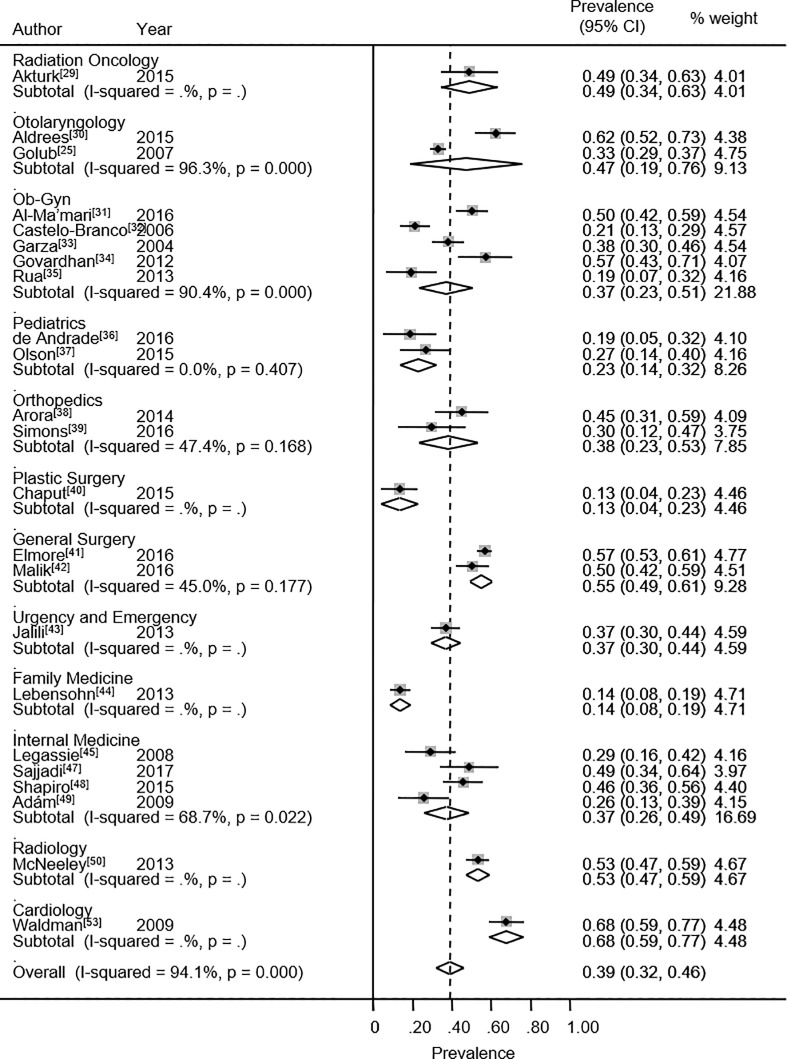
Emotional exhaustion. Forest plot of prevalence of high emotional exhaustion among medical and surgical residents.

The overall prevalence rate of high EE was 38.9% (95% CI: 31.8% - 46.0%). Twenty-three studies reported high EE. The heterogeneity chi-squared was 369.8% (p<0.001) with an *I*^2^ statistic of 94.1%. The specialties presenting the highest percentage values for high EE are general surgery (54.8%), otolaryngology (47.3%) and radiation oncology (48.9%) (Fig 4). In contrast, plastic surgery (63.5%),[40] and family medicine (58.3%)[44] exhibited the highest percentage values for low EE.

**Fig 5:**
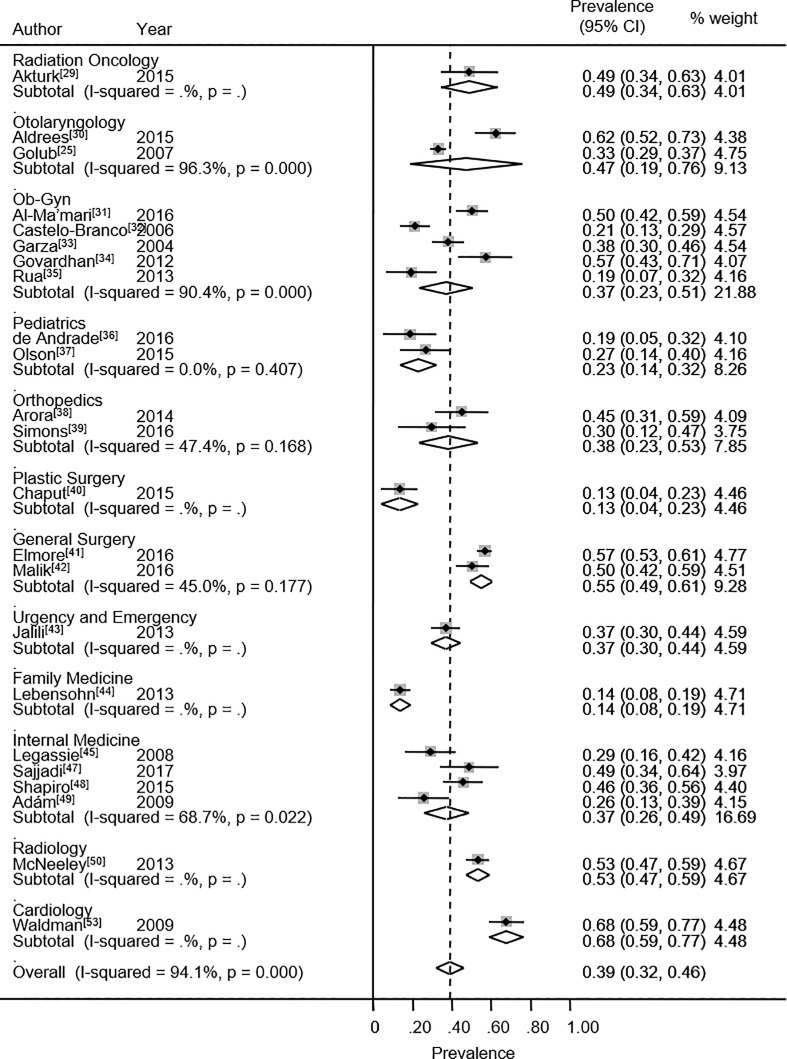
Personal accomplishment. Forest plot of prevalence of low personal accomplishment among medical and surgical residents.

The PA values demonstrated a different pattern. An overall value for low PA was equal to 34.3% (95% CI: 21.3% - 47.2%). Data was available from 20 studies. The heterogeneity chi-squared was 1348.6 (p<0.001) and *I*^2^ statistic was 98.6%. Internal medicine (59.2%);[45–49] plastic surgery (46.1%)[40] and urgency and emergency (46.1%)[43] had the residents with the lowest personal accomplishment values (Fig 5). However, radiation oncology (68.9%),[29] and cardiology (51.4%)[53] were the specialties with the highest personal accomplishment values.

Specialty, country and publication year were evaluated as sources of heterogeneity with meta-regression, but no statistically significant association was found. There was no evidence of publication bias or small study effects according to the Egger test. The estimated bias coefficient was 2.33 with a standard error of 2.18 (p = 0.30).

## Discussion

This systematic review and meta-analysis suggests that residents/interns in surgical/urgency (SU) specialties (general surgery, anesthesiology, obstetrics and gynecology and orthopedics) are those with the highest prevalence of burnout syndrome, confirming previous studies conducted on physicians after specialization[4] Brazilian study, evaluating 250 residents, and in agreement with our study, identified that surgical residences are positively associated with the syndrome. Additionally, having suffered a stressful event in the last 6 months was independently associated with the syndrome (this event may have been in the same environment as the medical residency).[54]

Obstetrics and gynecology and general surgery were also specialties with a high prevalence, according to a comprehensive search performed previously, suggesting that these specialties are consolidated residencies with the highest burnout rates.[21] A possible explanation for this fact could be the emergency routine, given that the resident is dealing directly with life-threatening situations and that there is an overload of shifts, conditions that are common to these specialties. Both analyses cited above also had the following low burnout syndrome specialties in common: otolaryngology, plastic surgery and neurology. These are predominantly clinical residencies, with substantially less shifts and more elective and non-urgent situations.

In regard to the subdimensions, obstetrics and gynecology was the only one of the three specialties with the highest DP values that also had a high overall burnout prevalence. Such high prevalence was confirmed in a previous meta-analysis,[55] which included 12 obstetrics and gynecology studies. Cardiology was the specialty with the highest DP, but as the single study on cardiology did not show overall burnout ratio, this specialty could not be considered in the high prevalence group. However, among the highest percentages of low DP specialties, only plastic surgery also had a low burnout syndrome level. This discrepancy could also be seen in subdimension EE: cardiology had high EE, but there was no description of the overall burnout prevalence, as was with the other two high specialties in this category. Regarding low EE levels, plastic surgery was the only specialty having low overall burnout rate. Despite no residence review studies having been found on this specialty, a systematic review[56] showed very similar results (burnout syndrome and its subdimensions) when analyzing plastic surgeons after residence. Therefore, this partial heterogeneity shows burnout as a complex and multi-dimensional syndrome, as one dimension can prevail over another. Therefore, its final definition proves to be the real interaction of its subdimensions.

Current scientific literature suggests some individual risk factors for the onset of the syndrome in physicians. As previously mentioned, chronic exposure to stress is the main risk factor.[57] In this sense, Drummond[58] states that the practice of clinical medicine itself would be an important factor, since the medical professional is in constant contact with sick people, with pain, patients, and their families. Along with this, there is the combination of great responsibility for the health of other human beings and the lack of dedication to one’s personal life: during medical training in residences, the doctor is not taught to balance his dedication to work with his/her private life. Curiously, in physicians working in urgent departments, rather than the severity of the patients, organizational factors such as impaired working relationships showed a greater association with burnout syndrome. Finally, certain sociodemographic characteristics appear to be risk factors in the medical population: a young age, female gender, negative marital status and high workload.[57]

Burnout syndrome in medical personal, however, appears not to be a problem generated in the medical residence. A Chinese systematic review including 33 studies found substantial burnout levels in medical students, with over 40% of Chinese medical students having more than moderate levels of burnout.[59] The higher levels were found in more senior students.

An American cross-sectional study evaluating the syndrome in medical students, verified its presence already in the pre-clinical years, with 71% of the students receiving the diagnosis. These future doctors, in a 2018 study, had already exhibited high values since the first year of medical school. Among the main risk factors, a cross-sectional study found that lack of confidence in the acquisition of medical knowledge, not seeing the course as a source of pleasure and discomfort with academic activities were positively correlated with the syndrome.[60]

Another systematic review performed recently evaluated specialists themselves (after residency), assessing 24 specialties. It showed an elevation in burnout incidence from 2011 to 2014 in all specialties, the most affected being emergency medicine, urology, orthopedics, internal medicine and anesthesiology.[4]

As stated previously,[61] burnout occurring in medical students from SU specialties has the potential to negatively affect at the personal and institutional level, which could result in a negative attitude, absenteeism, poor performance, as well as inducing medical errors. For each increase of one point in the depersonalization score (based on the MBI) there is an 11% increase in the likelihood of reporting a medical error.[5] On the other hand, for a one-point increase in the emotional exhaustion score there is a 5% increase in the likelihood of reporting an error. Even in the absence of medical errors, burnout culminates in the decline in the quality of medical care because both residents and practicing physicians with syndrome symptoms report a reduction in compassion at work, succinct conversations with patients, and other suboptimal patient care experiences.[14,62]

This situation demonstrates how the problem of burnout should be addressed in the contexts of training, education and work. First, it is necessary to recognize that the problem exists and what its impacts are. To evaluate the prevalence and intensity of the syndrome, the best option is to use validated instruments (MBI) and apply these to students, interns, residents and the medical specialists themselves, periodically. This will not only allow a view of the status, it will also allow for the measurement of the effectiveness of the measures adopted.

Strategies against burnout can be divided into preventive and therapeutic. In prevention, the actions must be concentrated on the risk factors mentioned above and, thus, the modification of the organizational structure and work processes, improvements in the relationship between the professional and the organization and the promotion of healthy behaviors in physicians (mainly resilience).[57]

In regard to therapeutic strategies, a 2018 systematic review of 13 studies summarizes the results of the research in proposing training, starting from medical graduation, or coping strategies, interpersonal skills, management of negative emotions and relaxation techniques.[63]

## Limitations of this review

Despite all the efforts deployed, this systematic review has certain limitations that should be considered when interpreting the results. Heterogeneity is a potential problem, since in the *I*^2^ statistic, there was a range from 87.6 to 98.6% in the summarized data. An explanation beyond meta-regression was attempted, but this analysis did not identify explanatory factors, probably due to the limited number of primary studies. Although, it is reasonable to consider that one possible explanation for this heterogeneity is that residents from different countries were included, and the different professional practices and cultures may have influenced the response to the MBI. Additionally, the study objective (including different medical specialties) may have concurred to increase this heterogeneity.

We minimized the likelihood of this issue by performing a careful search for published studies using explicit criteria for study inclusion, precise data extraction, and strict data analysis. In addition, as only cross-sectional studies were included, it is not possible to point out the main factors related to a higher prevalence of burnout syndrome in certain medical residences. Finally, it is an important fact that residency training differs from country to country and from program to program, and that program–or nation-specific data may not generalize well to a specialty-wide burnout rate.

Finally, this may lead to a critical interpretation of the meta-analysis and compromises any kind of generalization of the results, A larger number of standardized studies is needed, in order to reduce heterogeneity and, thus, allow for the application of future studies in another population of medical residents.

## Conclusions

Burnout syndrome was identified as having a high prevalence among surgical/urgency (SU) residencies, but is not a single specialty problem, it affects all the medical residencies in different ways. Additionally, it is a health care organization problem, damaging mainly the patients’ well-being and likely has a financial impact. This systematic review and meta-analysis, by analyzing its prevalence among different specialties, makes it possible to prioritize certain areas (such as SU specialties) in the fight against burnout and its consequences. It cannot be denied that clinical specialties are affected, but since they are not explicitly harmed by this syndrome, public health efforts should concentrate on where the problem is explicit. Therefore, health interventions, such as reductions in duty-hours, mindfulness training, psychiatry guided self-development groups and mantra induced meditation (Respiratory One Method), which have already been established and proposed [64] can contribute to the reduction of burnout in medical residencies such as general surgery, anesthesiology, obstetrics and gynecology and orthopedics.

## Supporting information

S1 File Prisma 2009 checklist. Preferred Reporting Items for Systematic Reviews and Meta-Analyses.
(PDF)

S2 File Search strategy. Mesh terms combination entered in databases.
(PDF)

S3 File Publication bias and small study effects. Graphic presentation of small study effects and publication bias.
(PDF)

S4 File Database. Compilation of all data collected during the research.
(XLSX)
