# Supplementary material for: Burnout syndrome among medical residents: A systematic review and meta-analysis
Source: PLoS One. 2018 Nov 12;13(11):e0206840. doi: 10.1371/journal.pone.0206840 (PMC6231624; doi:10.1371/journal.pone.0206840)
Supplement: S2 File — Mesh terms combination entered in databases. (PDF) [file pone.0206840.s002.pdf]

Detailed Search Criteria for https://www.ncbi.nlm.nih.gov/pubmed:

((((((medical resident) OR resident) OR residency training) OR residency) OR (internship and residency))) AND (((burnout) OR burnout syndrome) OR professional burnout

No filters.
