# Supplementary figures and images for: Burnout syndrome among medical residents: A systematic review and meta-analysis
Source: PLoS One. 2018 Nov 12;13(11):e0206840. doi: 10.1371/journal.pone.0206840 (PMC6231624; doi:10.1371/journal.pone.0206840)

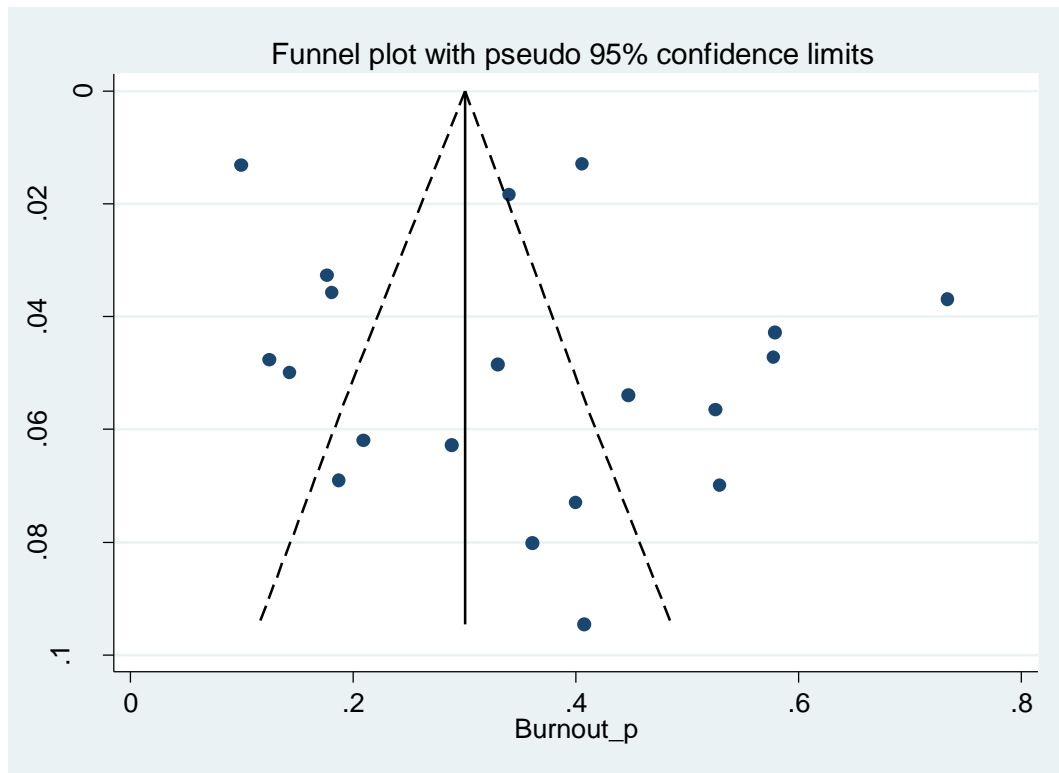

Supplement: S3 File — Graphic presentation of small study effects and publication bias. (PDF) [file pone.0206840.s003.pdf]
